# Serotonin uptake via plasma membrane monoamine transporter during myocardial ischemia‐reperfusion in the rat heart in vivo

**DOI:** 10.14814/phy2.14297

**Published:** 2019-11-28

**Authors:** Takashi Sonobe, Tsuyoshi Akiyama, Cheng‐Kun Du, James T. Pearson

**Affiliations:** ^1^ Department of Cardiac Physiology National Cerebral and Cardiovascular Center Research Institute Suita Osaka Japan

**Keywords:** 5‐HT, in vivo cardiac microdialysis, ischemia reperfusion injury, organic cation transporter, plasma membrane monoamine transporter, serotonin transporter

## Abstract

Serotonin (5‐HT) accumulates in the heart during myocardial ischemia and induces deleterious effects on the cardiomyocytes through receptor‐dependent and monoamine oxidase‐dependent pathways. We aimed to clarify the involvement of extra‐neuronal monoamine transporters in the clearance of 5‐HT during ischemia and reperfusion in the heart. Using a microdialysis technique in the anesthetized Wistar rat heart, we monitored myocardial interstitial 5‐HT and 5‐hydroxyindole acetic acid (5‐HIAA) concentration by means of electro‐chemical detection coupled with high‐performance liquid chromatography (HPLC‐ECD). Effects of inhibitors of the plasma membrane monoamine transporter (PMAT) and the organic cation transporter 3 (OCT3) (decynium‐22 and corticosterone) on the 5‐HT and 5‐HIAA concentrations during baseline, coronary occlusion, and reperfusion were investigated. Basal dialysate 5‐HT concentration were increased by local administration of decynium‐22, but not by corticosterone. Addition of fluoxetine, a serotonin transporter (SERT) inhibitor further increased the 5‐HT concentration upon during administration of decynium‐22. Decynium‐22 elevated the background level of 5‐HT during coronary occlusion and maintained 5‐HT concentration at a high level during reperfusion. Production of 5‐HIAA in the early reperfusion was significantly suppressed by decynium‐22. These results indicate that PMAT and SERT independently regulate basal level of interstitial 5‐HT, and PMAT plays a more important role in the clearance of 5‐HT during reperfusion. These data suggest the involvement of PMAT in the monoamine oxidase‐dependent deleterious pathway in the heart.

## Abstract 2

Plasma membrane monoamine transporter (PMAT) regulates interstitial level of serotonin in the heart. PMAT also contributes to uptake of accumulated serotonin into cells during reperfusion after the myocardial ischemia.

## Graphical abstract

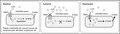

## 1 INTRODUCTION

Serotonin (5‐hydroxytryptamine, 5‐HT) accumulates in the heart during cardiac ischemia (Du et al., 2017; Shimizu et al., 2002; Sonobe, Akiyama, Du, Zhan, & Shirai, 2013). The increased myocardial interstitial level of 5‐HT directly induces myocardial cell injury through 5‐HT receptor‐dependent coronary vasoconstriction (Golino et al., 1989; Métais, Bianchi, Li, Simons, & Sellke, 2001), platelet aggregation (Willerson, Golino, Eidt, Campbell, & Buja, 1989), and overactivation of afferent cardiac sympathetic nerves (Fu & Longhurst, 2002; Longhurst, Tjen‐A‐Looi, & Fu, 2001). Meanwhile, it has been considered that the accumulated interstitial 5‐HT is taken up into cells via serotonin transporter (SERT) and then metabolized by monoamine oxidase (MAO) to produce 5‐hydroxyindole acetic acid (5‐HIAA) and hydrogen peroxide (Pizzinat, Copin, Vindis, Parini, & Cambon, 1999), which causes myocardial cell injury (Bianchi et al., 2005; Kaludercic, Carpi, Menabò, Di Lisa, & Paolocci, 2011). In order to develop novel therapeutic strategies to prevent the 5‐HT‐dependent cardiac injury, it is important to understand the mechanism by which 5‐HT is transported within the cardiac tissues during ischemia and reperfusion.

In our previous study using in vivo cardiac microdialysis technique in anesthetized rabbits (Sonobe et al., 2013) and rats (Du et al., 2017), we observed that local administration of SERT inhibitor fluoxetine had no effect on the clearance of accumulated 5‐HT in the heart. This data implied the contribution of fluoxetine‐resistant 5‐HT transporters, such as extra‐neuronal monoamine transporters (Furihata & Anzai, 2017), to remove 5‐HT from the interstitial space. Therefore, to clarify the contribution of 5‐HT transporters, which regulate 5‐HT level in the myocardial interstitium other than SERT, we examined effects of local administration of an inhibitor of plasma membrane monoamine transporter (PMAT) and/or organic cation transporter 3 (OCT3) on the basal level of interstitial 5‐HT in the in vivo heart. Furthermore, the effect of PMAT/OCT3 inhibition on the 5‐HT and 5‐HIAA kinetics was investigated during coronary occlusion and reperfusion.

## 2 MATERIALS AND METHODS

### 2.1 Animal preparation

Animal experiments were conducted in accordance with the Guiding Principles for the Care and Use of Animals in the Field of Physiological Sciences (Physiological Society of Japan), and approved by the Institutional Animal Care and Use Committee of National Cerebral and Cardiovascular Center Research Institute (No.17014 and 18017).

A total of 17 male Wistar rats (purchased from Japan SLC) weighing 466 ± 10 g (24 ± 1 week‐old) were used in this study. The rats were anesthetized by pentobarbital sodium (60 mg/kg, i.p.) supplemented with an analgesic agent (butorphanol, 0.25 mg/kg, i.p.). The rats were then quickly intubated and mechanically ventilated with room air mixed with oxygen. The animal's body temperature was maintained at around 37℃ using a heating pad and a lamp. A PE‐50 tubing was cannulated into the right jugular vein for continuous infusion of pentobarbital (20 mg kg^‐1^ h^‐1^) and butorphanol (0.1 mg kg^‐1^ h^‐1^) and a similar tubing was inserted into the right carotid artery for monitoring arterial pressure. Arterial blood pressure and electrocardiogram were monitored via a signal transducer and the signals were digitized by using an A/D data acquisition system (Power Lab 16/35, AD Instruments, Inc.). Heart rate was determined by peak detection of the electrocardiogram on LabChart8 software (AD Instruments, Inc.).

### 2.2 Cardiac microdialysis technique

**Figure 1:**
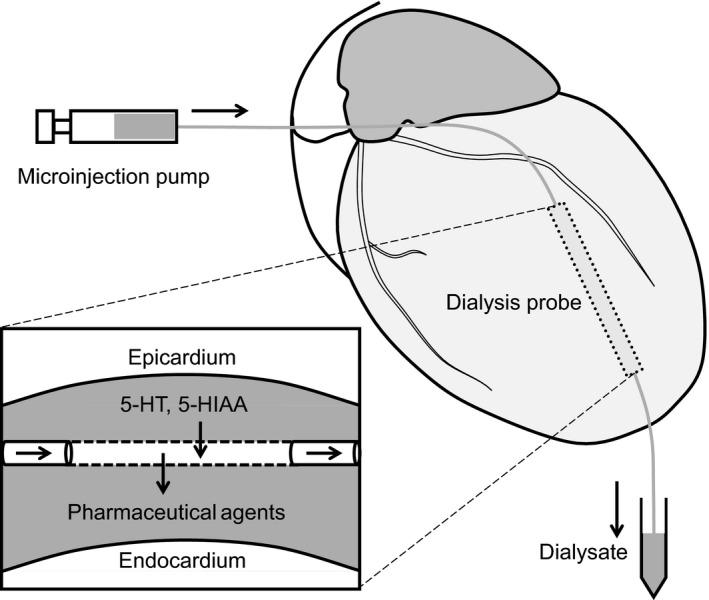
Schematic illustration of the cardiac microdialysis. A dialysis probe was implanted transversely into the lateral wall of the left ventricle. Interstitial 5‐HT and 5‐HIAA diffuse into perfusate across the dialysis membrane, and pharmaceutical agents diffuse into the interstitial space according to the concentration gradient. Dialysate from the myocardial interstitium was sampled and then analyzed using HPLC‐ECD. A silk suture was passed around the coronary artery and occluded as necessary

Details of the cardiac microdialysis technique have been described previously (Akiyama, Yamazaki, & Ninomiya, 1991; Sonobe, Akiyama, Du, Zhan, & Shirai, 2014). Briefly, with the rat in a lateral position, left thoracotomy was performed and a dialysis probe was implanted transversely into the lateral wall of the left ventricle (Figure 1). A 4–0 silk suture was passed around the left coronary artery for coronary occlusion in the protocol of ischemia‐reperfusion. The implanted microdialysis probe was continuously perfused with Ringer's solution or Ringer's solution containing pharmacological agent at a rate of 2 µl/min using a microinjection pump (CMA/100, Carnegie Medicine). Sampling time of dialysate was 7.5 or 15 min yielding 15 or 30 µl of dialysate, respectively. The first dialysate sampling was started from at least 2 hr after the probe implantation. Serotonin concentration in the dialysate sample was measured by electro‐chemical detection coupled with high performance liquid chromatography (HPLC‐ECD, Eicom) as described previously (Du et al., 2017, 2014; Sonobe et al., 2013).

### 2.3 Experimental protocols

**Figure 2:**
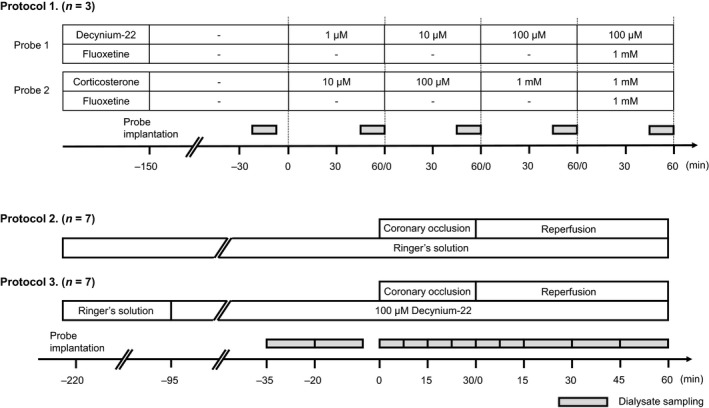
Time course of the experimental protocols. Protocol 1) The implanted probe was perfused with Ringer's solution containing various concentrations of decynium‐22 or corticosterone. Fluoxetine was co‐administered with the highest concentration of decynium‐22 or corticosterone. An aliquot of dialysate was sampled at baseline and 45–60 min into each perfusion period for 15 min. Protocol 2 and 3) Aliquots of dialysate were sampled before, during, and after the coronary occlusion as shown in the timeline. In the protocol 3, the perfusate was changed to the solution containing 100 µM decynium‐22 at 60 min before the baseline sampling

The time course of the experiments and dialysate sampling time points are shown in Figure 2.

#### 2.3.1 Protocol 1

Effect of local inhibition of OCT3 and/or PMAT on the basal concentration of myocardial interstitial 5‐HT was investigated in three rats. In this protocol, three rats were used, and two dialysis probes were implanted in the heart with at least 5 mm of distance in between them. After the baseline dialysate sampling, the one probe was perfused with Ringer's solution containing decynium‐22, a PMAT/OCT3 inhibitor (Duan et al., 2015; Feng et al., 2005; Schömig, Babin‐Ebell, & Russ, 1993; Wang, 2016). During the last 10 min into 60‐min of the perfusion, dialysate sample was collected for 5‐HT measurement. Three different concentrations of decynium‐22 (1, 10, and 100 µM) were tested continuously, and 100 µM decynium‐22 mixed with 1 mM fluoxetine (SERT inhibitor) perfused during the fourth 60‐min. At the same time, the other probe was perfused with Ringer's solution containing corticosterone (10, 100 µM, and 1 mM), an inhibitor of OCT3 (Engel & Wang, 2005; Hayer‐Zillgen, Brüss, & Bönisch, 2002). Time course of dialysate sampling was similar to that for decynium‐22.

#### 2.3.2 Protocol 2

Contribution of OCT3/PMAT to interstitial 5‐HT clearance during myocardial ischemia and reperfusion was investigated. In seven rats (control group, *n* = 7), the coronary artery was occluded for 30 min and then re‐perfused for 60 min. The baseline dialysate was sampled before the coronary occlusion, and continuously sampled during the coronary occlusion and reperfusion (Figure 2).

#### 2.3.3 Protocol 3

In additional rats (decynium‐22 group, *n* = 7), the perfusate was switched from Ringer's solution to 100 µM of decynium‐22 solution at one hour before the baseline dialysate sampling, allowing local administration of decynium‐22 into the myocardium during coronary occlusion. The concentration and stabilization time for perfusing decynium‐22 was chosen based on results from our preliminary experiments and the experiments in protocol 1. Time course of dialysate sampling during 30 min of coronary occlusion and 60 min of reperfusion was the same as in the control group of animals.

### 2.4 Drugs

Decynium‐22 was purchased from Tocris Bioscience, corticosterone was purchased from Sigma‐Aldrich, and fluoxetine was purchased from FUJIFILM Wako Pure Chemical. Stock solution of decynium‐22 and corticosterone was prepared in DMSO at concentration of 1 mM and 10 mM, respectively. Stock solution of 10 mM fluoxetine was prepared in distilled water. These stock solutions were stored at −30℃ and were diluted in Ringer's solution freshly before the experiments.

### 2.5 Statistical analysis

All statistical analyses were conducted with GraphPad Prism 5 (GraphPad Software). All results were presented as means ± SE, unless otherwise stated. Time course changes in dialysate 5‐HT and 5‐HIAA concentration were compared by one‐way repeated measures ANOVA followed by a Bonferroni's test. Differences between the groups were compared by two‐way repeated measures ANOVA followed by a Bonferroni's test. Differences were considered significant at *p* < .05.

## 3 RESULTS

### 3.1 Time course of heart rate and mean arterial blood pressure

Heart rate and mean arterial blood pressure in animals during the experiments are shown in Table 1. Baseline heart rate and mean arterial blood pressure were similar among the groups. In the protocol 1, local administration of decynium‐22 or corticosterone had no systemic effects on the time course changes in heart rate and mean arterial pressure, indicating that both the administered decynium‐22 and corticosterone locally affected the myocardium around the implanted dialysis probe. In the protocol 2, about 85% of animals presented moderate to severe ventricular tachycardia or ventricular fibrillation at 7–10 min into the coronary occlusion. In a few cases, mean arterial blood pressure dropped to less than 30 mmHg, and direct cardiac compression was required. Local administration of decynium‐22 did not affect the occurrence of such ventricular tachycardia or ventricular fibrillation and the time course changes in heart rate and mean arterial blood pressure throughout the experiments.

**Table 1:** Time course of heart rate and mean arterial blood pressure

Protocol 1.	Baseline	1st 45–60 min	2nd 45–60 min	3rd 45–60 min	4th 45–60 min
HR (bpm)	364 ± 27	375 ± 23	374 ± 20	372 ± 26	382 ± 24
MABP (mmHg)	91 ± 9	89 ± 7	83 ± 6	82 ± 4	91 ± 10

Protocol 2.	Baseline	Ischemia				Reperfusion				
		0–7.5 min	7.5–15.0 min	15.0–22.5 min	22.5–30.0 min	0–7.5 min	7.5–15.0 min	15–30 min	30–45 min	45–60 min
HR (bpm)	359 ± 8	359 ± 8	362 ± 9	379 ± 8	380 ± 9	377 ± 6	376 ± 6	378 ± 5	379 ± 5	375 ± 7
MABP (mmHg)	88 ± 5	68 ± 1*	73 ± 5*	87 ± 5	84 ± 5	70 ± 2*	71 ± 2*	76 ± 3	77 ± 3	75 ± 3*

Protocol 3.	Baseline	Ischemia				Reperfusion				
		0–7.5 min	7.5–15.0 min	15.0–22.5 min	22.5–30.0 min	0–7.5 min	7.5–15.0 min	15–30 min	30–45 min	45–60 min
HR (bpm)	367 ± 4	364 ± 6	370 ± 11	386 ± 5	389 ± 5	384 ± 5	385 ± 5	380 ± 4	379 ± 4	373 ± 4
MABP (mmHg)	86 ± 5	68 ± 5*	72 ± 7*	76 ± 5	73 ± 5*	65 ± 2*	68 ± 3*	70 ± 4*	71 ± 3*	70 ± 3*

Note

Values are shown in mean ± SE. In the protocol 1 (*n* = 3, see Figure 1), HR and MABP were stable throughout the experiment. In the protocol 2 (*n* = 7) and 3 (*n* = 7), there were significant changes in MABP (*p* < .05 vs. Baseline) caused by myocardial ischemia‐reperfusion; however, there were no significant differences between the protocol 2 and 3 at any corresponding time points.

* *p* < .05, versus Baseline. One‐way repeated ANOVA followed by Bonferroni's post‐test.

### 3.2 Effect of decynium‐22 and corticosterone on the basal 5‐HT and 5‐HIAA concentration in the heart

**Figure 3:**
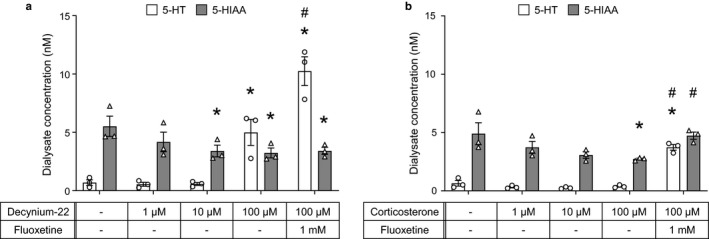
Effects of local administration of decynium‐22 or corticosterone on the baseline dialysate 5‐HT concentration (*n* = 3). Decynium‐22 significantly increased 5‐HT concentration at 100 µM (a), meanwhile corticosterone had no effect on 5‐HT concentration (b). Addition of fluoxetine in the perfusate further increased the 5‐HT concentration in both decynium‐22 and corticosterone treatment (a and b). Data are shown in individual data points and mean ± SE. *p < .05, versus baseline (−/−). One‐way repeated ANOVA followed by Bonferroni's post‐test. #*p* < .05, versus (100 µM decynium‐22 or 1 mM corticosterone alone). One‐way repeated ANOVA followed by Bonferroni's post‐test

Baseline concentration of dialysate 5‐HT and 5‐HIAA was 0.7 ± 0.2 nM and 5.5 ± 0.9 nM in dialysate from the probe perfused with decynium‐22, respectively (Figure 3a). Local administration of 100 µM decynium‐22 significantly increased dialysate 5‐HT concentration (5.0 ± 1.1 nM, *p* < .05 vs. baseline) but decreased dialysate 5‐HIAA concentration (3.2 ± 0.4 nM, *p* < .05 vs. baseline). Lower concentration of decynium‐22 (1 and 10 µM) did not change the baseline 5‐HT concentration, while 5‐HIAA concentration started to decrease with 10 µM decynium‐22 (*p* < .05). Co‐administration of 100 µM decynium‐22 and 1 mM fluoxetine further increased dialysate 5‐HT concentration compared to that in decynium‐22 alone (10.2 ± 1.2 nM, *p* < .05 vs. baseline, and vs. 100 µM decynium‐22 alone), however, there was no additional effect on dialysate 5‐HIAA concentration.

Baseline 5‐HT and 5‐HIAA concentrations in dialysate from the probe perfused with corticosterone were similar to that in decynium‐22 group (0.6 ± 0.2 and 4.9 ± 0.9 nM, respectively) (Figure 3b). In contrast with the effect of decynium‐22 on the 5‐HT concentration, local administration of corticosterone did not change basal level of 5‐HT (Figure 3b). Significant increase in 5‐HT concentration was found only when fluoxetine was co‐administered (3.7 ± 0.2 nM, *p* < .05 vs. baseline, and vs. 1 mM corticosterone alone). Dialysate 5‐HIAA concentration decreased and indicated a significant difference in the condition of 1 mM corticosterone (2.7 ± 0.1 nM, *p* < .05 vs. baseline). The dialysate 5‐HIAA concentration returned to baseline level after the administration of 1 mM corticosterone and 1 mM fluoxetine, and it was significantly high compared to the level at 1 mM corticosterone alone (4.7 ± 0.3 nM, *p* < .05 vs. 1 mM corticosterone alone).

### 3.3 Effect of decynium‐22 on 5‐HT and 5‐HIAA concentrations during coronary occlusion

#### 3.3.1 Control group

**Figure 4:**
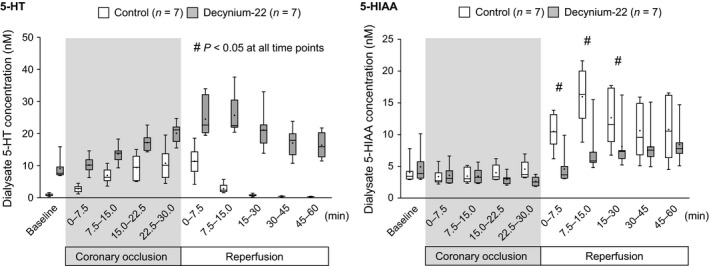
Effect of decynium‐22 on dialysate 5‐HT and 5‐HIAA concentration during coronary occlusion and reperfusion. In control group (*n* = 7), 5‐HT increased during coronary occlusion and then immediately decreased after reperfusion, while 5‐HIAA kept the baseline level during coronary occlusion and abruptly increased after reperfusion. In decynium‐22 group (*n* = 7), background level of 5‐HT rose at baseline and during coronary occlusion and did not return to the baseline level after reperfusion. At baseline and during coronary occlusion 5‐HIAA level was not affected by decynium‐22, however, the increase in 5‐HIAA in the early reperfusion was significantly suppressed. Data are presented as box‐whisker plots. The horizontal line in the box indicates the median, and the dot in the box indicates the mean. #*p* < .05, versus Control. Two‐way repeated ANOVA followed by Bonferroni's post‐test

Dialysate 5‐HT concentration at baseline was 0.9 ± 0.2 nM. Similar to our previous finding, coronary occlusion increased dialysate 5‐HT concentration (Figure 4). The 5‐HT concentration started to increase at 7.5–15 min (*p* < .05 vs. baseline) and reached 10.8 ± 1.9 nM at 22.5–30 min into the coronary occlusion (*p* < .05 vs. baseline). Meanwhile, dialysate 5‐HIAA concentration was maintained at baseline level (4.0 ± 0.7 nM) during the 30 min of coronary occlusion.

#### 3.3.2 Decynium‐22 group

Baseline concentration of 5‐HT was significantly elevated as we demonstrated above (9.1 ± 1.2 nM, *p* < .05 vs. control) due to the local administration of 100 µM decynium‐22. The 5‐HT concentration started to increase at 7.5–15 min (*p* < .05 vs. baseline) and then reached 20.1 ± 1.2 nM at 22.5–30 min into the coronary occlusion (*p* < .05 vs. baseline). Dialysate concentration of 5‐HIAA did not change during the coronary occlusion.

The dialysate 5‐HT concentration was significantly higher than that in the control group at all time points during the coronary occlusion (*p* < .05), meanwhile there was no significant difference in the dialysate 5‐HIAA concentration between the control and the decynium‐22 group.

### 3.4 Effect of decynium‐22 on the 5‐HT and 5‐HIAA concentration during reperfusion

#### 3.4.1 Control group

Dialysate 5‐HT concentration reached a peak within the first sampling of the reperfusion period (11.4 ± 1.7 nM at 0–7.5 min reperfusion, *p* < .05 vs. baseline). Thereafter, the dialysate 5‐HT concentration largely decreased and returned to baseline level at 15–30 min into the reperfusion period, and it remained lower up until 60 min of the reperfusion period. Dialysate 5‐HIAA concentration abruptly increased when the reperfusion was initiated (*p* < .05). The dialysate 5‐HIAA concentration reached a peak within 7.5–15 min into reperfusion (16.0 ± 1.8 nM, *p* < .05 vs. baseline), and was maintained at a higher level during rest of the reperfusion period (Figure 4).

#### 3.4.2 Decynium‐22 group

The peak of dialysate 5‐HT concentration was found at 7.5–15 min into the reperfusion (25.7 ± 2.3 nM, *p* < .05 vs. baseline). In contrast to 5‐HT kinetics in the control group, dialysate 5‐HT concentration was kept at a higher level (*p* < .05 vs. control), and did not return to baseline level, at least by 60 min of reperfusion. Dialysate 5‐HIAA concentration gradually increased and indicated a significantly higher value after 15 min of reperfusion (*p* < .05 vs. baseline).

The 5‐HT and 5‐HIAA kinetics were dramatically different during the reperfusion period between the groups. The 5‐HT concentration was elevated in the decynium‐22 group at the all timepoints (*p* < .05 vs. control) while the 5‐HIAA concentration was significantly lower in the decynium‐22 group during the early phase of the reperfusion (*p* < .05 vs. control, 0–30 min of reperfusion).

## 4 DISCUSSION

Inhibition of extra‐neuronal monoamine transporters by decynium‐22 but not by corticosterone increased interstitial 5‐HT concentration in the heart in vivo. This effect was independent from fluoxetine‐sensitive increase in 5‐HT. During myocardial ischemia, administration of decynium‐22 did not change the kinetics of 5‐HT accumulation although the concentration was above the normal level during coronary occlusion. In contrast, 5‐HT clearance during reperfusion was largely decreased in the presence of decynium‐22. Moreover time course changes in 5‐HIAA concentration indicated that 5‐HIAA production, that is, 5‐HT metabolism, was depressed in the early phase of reperfusion by decynium‐22. These data suggest that PMAT plays an important role in the MAO‐dependent pathway leading to cellular damage in the heart during myocardial ischemia‐reperfusion.

### 4.1 Regulation of basal level of interstitial 5‐HT and 5‐HIAA

Myocardial interstitial 5‐HT concentration increased during local administration of decynium‐22, which is known to inhibit not only PMAT but also OCTs (Duan et al., 2015; Feng et al., 2005; Schömig et al., 1993; Wang, 2016), and then stabilized about at 60 min into the administration. This increase in 5‐HT was not observed during administration of corticosterone, which inhibits OCT3 more selectively (Duan et al., 2015; Wang, 2016). Although it has been reported that both PMAT and OCT3 take up 5‐HT into cells, for example vascular smooth muscle cells from the human brain (Li et al., 2013), OCT3 is less likely to contribute to the maintenance of the basal level of 5‐HT in the heart compared to PMAT. The expression of PMAT has been observed in the cardiomyocytes from rats and mice (Barnes et al., 2006; Gergs et al., 2017), however, OCT3 expression is relatively weak in the cardiomyocytes (Gergs et al., 2017; Grube et al., 2011; Solbach, Grube, Fromm, & Zolk, 2011). These previous studies support our viewpoint of basal regulation of interstitial 5‐HT via PMAT in the heart.

Additional administration of fluoxetine mixed with decynium‐22 or corticosterone induced further increase in the interstitial 5‐HT concentration regardless of the level of 5‐HT before the fluoxetine administration. In keeping with similar findings in the brain (Hagan, Schenk, & Neumaier, 2011; Horton et al., 2013), our results suggest that low‐affinity and high‐capacity PMAT (Engel, Zhou, & Wang, 2004) and high‐affinity and low‐capacity SERT independently but synergistically modulate the basal level of interstitial 5‐HT in the heart. Although SERT found in the bovine heart (Mortensen, Kristensen, Rudnick, & Wiborg, 1999) and fetal heart (Sari & Zhou, 2003) has been reported, there is no strong evidence of SERT expression in the heart. A major source of 5‐HT uptake via SERT might be platelets, which has been well established to express SERT (Lesch, Wolozin, Murphy, & Reiderer, 1993) more so than cardiomyocytes.

Meanwhile, 5‐HIAA level was not changed by the addition of fluoxetine in the presence of decynium‐22 but increased in the presence of corticosterone, indicating that 5‐HT uptake followed by 5‐HIAA production were not inhibited by corticosterone. This data also supports our view that PMAT, rather than OCT3 is involved in regulation of the basal 5‐HT turnover in the heart.

### 4.2 Contribution of PMAT to 5‐HT uptake during ischemia

Throughout the ischemic period, we observed that dialysate 5‐HT concentration was about two times higher in the condition of decynium‐22 compared to the control condition, but the quantity of 5‐HT increase from the baseline (delta increase in 5‐HT) was quite similar to that in the control group. Uptake of 5‐HT via PMAT or OCTs has been known as uptake‐2, which is non‐energy dependent mechanism to transport monoamines according to a concentration gradient (Dahlin, Xia, Kong, Hevner, & Wang, 2007; Gergs et al., 2017), therefore it had been thought that 5‐HT uptake via uptake‐2 was not affected by the ischemia. However, our results indicate that the inhibition of PMAT/OCT3 did not change the rate of accumulation of 5‐HT during ischemia and suggests that PMAT/OCT3 was not contributing to the clearance of 5‐HT during ischemia. It remains unclear why the uptake via PMAT was limited during this period even though the driving force to produce 5‐HT influx, that is, the interstitial 5‐HT concentration, increased due to the ischemia.

In spite of the elevated 5‐HT level in the presence of decynium‐22, 5‐HIAA production during the ischemic period was similar to that in the control condition. This indicates that 5‐HT turnover was already depressed by pre‐administration of decynium‐22 before initiating the coronary occlusion.

### 4.3 Contribution of PMAT to 5‐HT uptake during reperfusion

After the initiation of reperfusion, accumulated 5‐HT did not return to the baseline level following inhibition of PMAT, in contrast to the immediate decrease in the control condition. This effect of PMAT inhibition was also confirmed by the suppression of 5‐HIAA production in the first 30 min of reperfusion. Furthermore, estimated 5‐HT turnover rate during reperfusion indicated that turnover of the accumulated 5‐HT was dramatically suppressed by the inhibition of PMAT. This effect was not observed by inhibition of SERT (Du et al., 2017), moreover it was similar to the effect of MAO inhibition by pargyline as we reported previously (Du et al., 2017). We have also demonstrated MAO‐dependent production of hydroxyl radical in the heart during ischemia and reperfusion (Inagaki et al., 2016). These findings suggest that PMAT is involved in the MAO‐dependent deleterious effects in the reperfusion‐induced cellular injury (Bianchi et al., 2005; Kaludercic et al., 2011).

The previous studies on non‐cardiomyocytes might support the view of the contribution of PMAT during the early reperfusion. It has been reported that SERT‐dependent 5‐HT uptake decreases with increasing extracellular 5‐HT concentration in synaptosomes (Hagan et al., 2011), and SERT‐independent 5‐HT uptake seemed to be predominant when the extracellular 5‐HT concentration increased (Hagan et al., 2011). A similar mechanism has also been suggested in platelets as increasing extracellular 5‐HT level might decrease SERT density on the plasma membrane (Brenner et al., 2007). These findings suggest that if 5‐HT accumulates in the interstitial space more than the capacity level of SERT due to the ischemia, reuptake of 5‐HT via SERT slows down, and instead, 5‐HT transport via PMAT becomes predominant in the clearance of 5‐HT.

### 4.4 Limitations

We used decynium‐22 (100 µM highest dose) to inhibit fluoxetine‐resistant 5‐HT transporters. This compound has been reported to inhibit PMAT and OCT3 with equal potency (Ki; 0.1 mM; Engel & Wang, 2005; Hayer‐Zillgen et al., 2002; Wang, 2016). We also used corticosterone (1 mM highest dose), which has been reported to be more selective toward OCTs with IC50 values in 0.29–34 mM for human OCT1‐3 (Hayer‐Zillgen et al., 2002), to differentiate the contribution of PMAT and OCTs on 5‐HT transport. Although our finding suggested that PMAT was plausibly involved in the 5‐HT transport in the heart, selective and reliable PMAT inhibitors (Duan et al., 2015) are required for further study. In addition, the highest concentration of decynium‐22, corticosterone, and fluoxetine might have non‐specific side effects on the cardiac cells. Microdialysis technique allows us to administer pharmacological agents through the dialysis membrane according to a concentration gradient. It requires typically 100 times higher drug concentration compared to direct administration in pharmacological studies using in vitro perfused organ or tissue, to affect cells near by the implanted dialysis probe. For instance, a similar concentration of inhibitor was used in a previously published microdialysis study. Feng et al. (2005) used microdialysis technique in rat brain and administered 10 ~ 100 µM of decynium‐22. They reported behavioral changes due to increased level of 5‐HT only when 100 µM decynium‐22 was administered. Therefore, we considered that high concentration of inhibitors, such as 100 µM of decysnium‐22 was necessary to inhibit 5‐HT transporters when it was administered through the dialysis membrane.

The cardiac microdialysis technique allows administering pharmacological agents during ischemia and reperfusion locally in the heart without major effect on global cardiac function or outcome of ischemia reperfusion injury. It is possible that systemic (e.g., intravenous) administration of decynium‐22 alters the outcome of ischemia reperfusion injury; however, serious side effects may also present through the increase in global 5‐HT level leading to activation of 5‐HT receptor‐dependent deleterious effects. Since our findings do not directly connect inhibition of 5‐HT uptake with prevention of myocardial infarction yet, further studies are required to establish a therapeutic approach through 5‐HT regulation in the heart.

## 5 Summary

**Figure 5:**
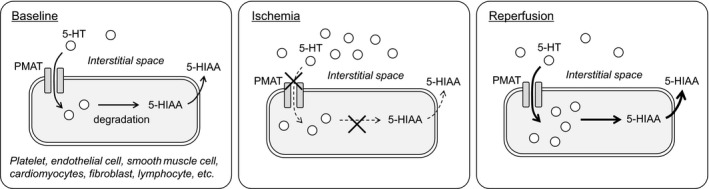
Putative pathway involving in the 5‐HT metabolism during ischemia and reperfusion in the heart. In a baseline condition (left), PMAT takes up 5‐HT into cells including cardiomyocytes, endothelial cells, and fibroblast cells to maintain background level of interstitial 5‐HT lower. During ischemia (middle), PMAT keeps the 5‐HT uptake rate while interstitial 5‐HT increases. In spite of the increase in intracellular 5‐HT, interstitial 5‐HIAA does not change due to reduced 5‐HT degradation in the cells. During reperfusion (right), high‐capacity transporter PMAT contributes to the clearance of the increased interstitial 5‐HT, meanwhile reoxygenation may accelerate 5‐HT degradation, therefore, intracellular 5‐HT is metabolized to 5‐HIAA

Based on these evidences discussed above, a putative mechanism of 5‐HT uptake into cells, and production of 5‐HIAA is summarized in Figure 5. During cardiac ischemia, 5‐HT accumulated in the ischemic region. In this period, 5‐HT uptake into cardiomyocytes via PMAT is somehow limited and activity of oxygen‐dependent 5‐HT degradation is suppressed, thus 5‐HIAA production decreased. However once blood/oxygen supply is re‐initiated by reperfusion, the accumulated 5‐HT is rapidly removed by uptake via high‐capacity PMAT. The 5‐HT taken into the cardiomyocytes is then degraded into 5‐HIAA and hydrogen peroxide, which induces cellular damage.

In conclusion, extra‐neuronal monoamine transporter PMAT and SERT independently contribute to regulate the basal concentration of interstitial 5‐HT in the heart in normal conditions. Meanwhile, PMAT plays a more important role in 5‐HT clearance followed by 5‐HT degradation in the cells during myocardial ischemia‐reperfusion.

## CONFLICT OF INTEREST

No conflicts of interest, financial, or otherwise, are declared by the authors.

## AUTHOR CONTRIBUTION

T.S. performed experiments, prepared figures, and drafted manuscript. T.S. and T.A. conceived and designed research, analyzed data, and interpreted results of experiments. T.S., T.A., C.K.D., and J.T.P. edited and revised manuscript, approved final version of manuscript.

## References

[phy214297-bib-0001] Akiyama, T. , Yamazaki, T. , & Ninomiya, I. (1991). In vivo monitoring of myocardial interstitial norepinephrine by dialysis technique. American Journal of Physiology, 261, H1643–H1647. 1951751 10.1152/ajpheart.1991.261.5.H1643

[phy214297-bib-0002] Barnes, K. , Dobrzynski, H. , Foppolo, S. , Beal, P. R. , Ismat, F. , Scullion, E. R. , … Baldwin, S. A. (2006). Distribution and functional characterization of equilibrative nucleoside transporter‐4, a novel cardiac adenosine transporter activated at acidic pH. Circulation Research, 99, 510–519. 16873718 10.1161/01.RES.0000238359.18495.42

[phy214297-bib-0003] Bianchi, P. , Kunduzova, O. , Masini, E. , Cambon, C. , Bani, D. , Raimondi, L. , … Parini, A. (2005). Oxidative stress by monoamine oxidase mediates receptor‐independent cardiomyocyte apoptosis by serotonin and postischemic myocardial injury. Circulation, 112, 3297–3305. 16286591 10.1161/CIRCULATIONAHA.104.528133

[phy214297-bib-0004] Brenner, B. , Harney, J. T. , Ahmed, B. A. , Jeffus, B. C. , Unal, R. , Mehta, J. L. , & Kilic, F. (2007). Plasma serotonin levels and the platelet serotonin transporter. Journal of Neurochemistry, 102, 206–215. 17506858 10.1111/j.1471-4159.2007.04542.x PMC3041643

[phy214297-bib-0005] Dahlin, A. , Xia, L. , Kong, W. , Hevner, R. , & Wang, J. (2007). Expression and immunolocalization of the plasma membrane monoamine transporter in the brain. Neuroscience, 146, 1193–1211. 17408864 10.1016/j.neuroscience.2007.01.072 PMC2683847

[phy214297-bib-0006] Du, C. K. , Zhan, D. Y. , Akiyama, T. , Inagaki, T. , Shishido, T. , Shirai, M. , & Pearson, J. T. (2017). Myocardial interstitial levels of serotonin and its major metabolite 5‐hydroxyindole acetic acid during ischemia‐reperfusion. American Journal of Physiology‐Heart and Circulatory Physiology, 312, H60–H67. 27793854 10.1152/ajpheart.00471.2016

[phy214297-bib-0007] Du, C.‐K. , Zhan, D.‐Y. , Akiyama, T. , Sonobe, T. , Inagaki, T. , & Shirai, M. (2014). Myocardial interstitial serotonin and its major metabolite, 5‐hydroxyindole acetic acid levels determined by microdialysis technique in rat heart. Life Sciences, 117, 33–39. 25277944 10.1016/j.lfs.2014.09.019

[phy214297-bib-0008] Duan, H. , Hu, T. , Foti, R. S. , Pan, Y. , Swaan, P. W. , & Wang, J. (2015). Potent and selective inhibition of plasma membrane monoamine transporter by HIV protease inhibitors. Drug Metabolism and Disposition, 43, 1773–1780. 26285765 10.1124/dmd.115.064824 PMC4613949

[phy214297-bib-0009] Engel, K. , & Wang, J. (2005). Interaction of organic cations with a newly identified plasma membrane monoamine transporter. Molecular Pharmacology, 68, 1397–1407. 16099839 10.1124/mol.105.016832

[phy214297-bib-0010] Engel, K. , Zhou, M. , & Wang, J. (2004). Identification and characterization of a novel monoamine transporter in the human brain. Journal of Biological Chemistry, 279, 50042–50049. 15448143 10.1074/jbc.M407913200

[phy214297-bib-0011] Feng, N. , Mo, B. , Johnson, P. L. , Orchinik, M. , Lowry, C. A. , & Renner, K. J. (2005). Local inhibition of organic cation transporters increases extracellular serotonin in the medial hypothalamus. Brain Research, 1063, 69–76. 16266691 10.1016/j.brainres.2005.09.016

[phy214297-bib-0012] Fu, L. W. , & Longhurst, J. C. (2002). Activated platelets contribute to stimulation of cardiac afferents during ischaemia in cats: Role of 5‐HT(3) receptors. Journal of Physiology, 544, 897–912. 12411532 10.1113/jphysiol.2002.023374 PMC2290632

[phy214297-bib-0013] Furihata, T. , & Anzai, N. (2017). Functional expression of organic ion transporters in astrocytes and their potential as a drug target in the treatment of central nervous system diseases. Biological and Pharmaceutical Bulletin, 40, 1153–1160. 28768996 10.1248/bpb.b17-00076

[phy214297-bib-0014] Gergs, U. , Jung, F. , Buchwalow, I. B. , Hofmann, B. , Simm, A. , Treede, H. , & Neumann, J. (2017). Pharmacological and physiological assessment of serotonin formation and degradation in isolated preparations from mouse and human hearts. American Journal of Physiology‐Heart and Circulatory Physiology, 313, H1087–H1097. 28916638 10.1152/ajpheart.00350.2017

[phy214297-bib-0015] Golino, P. , Ashton, J. H. , Buja, L. M. , Rosolowsky, M. , Taylor, A. L. , McNatt, J. , … Willerson, J. T. (1989). Local platelet activation causes vasoconstriction of large epicardial canine coronary arteries in vivo. Thromboxane A2 and serotonin are possible mediators. Circulation, 79, 154–166. 2910540 10.1161/01.cir.79.1.154

[phy214297-bib-0016] Grube, M. , Ameling, S. , Noutsias, M. , Köck, K. , Triebel, I. , Bonitz, K. , … Kroemer, H. K. (2011). Selective regulation of cardiac organic cation transporter novel type 2 (OCTN2) in dilated cardiomyopathy. American Journal of Pathology, 178, 2547–2559. 21641380 10.1016/j.ajpath.2011.02.020 PMC3124333

[phy214297-bib-0017] Hagan, C. E. , Schenk, J. O. , & Neumaier, J. F. (2011). The contribution of low‐affinity transport mechanisms to serotonin clearance in synaptosomes. Synapse (New York, N. Y.), 65, 1015–1023. 10.1002/syn.20929 PMC3149756 21437992

[phy214297-bib-0018] Hayer‐Zillgen, M. , Brüss, M. , & Bönisch, H. (2002). Expression and pharmacological profile of the human organic cation transporters hOCT1, hOCT2 and hOCT3. British Journal of Pharmacology, 136, 829–836. 12110607 10.1038/sj.bjp.0704785 PMC1573414

[phy214297-bib-0019] Horton, R. E. , Apple, D. M. , Owens, W. A. , Baganz, N. L. , Cano, S. , Mitchell, N. C. , … Daws, L. C. (2013). Decynium‐22 enhances SSRI‐induced antidepressant‐like effects in mice: Uncovering novel targets to treat depression. Journal of Neuroscience, 33, 10534–10543. 23785165 10.1523/JNEUROSCI.5687-11.2013 PMC3685842

[phy214297-bib-0020] Inagaki, T. , Akiyama, T. , Du, C. K. , Zhan, D. Y. , Yoshimoto, M. , & Shirai, M. (2016). Monoamine oxidase‐induced hydroxyl radical production and cardiomyocyte injury during myocardial ischemia‐reperfusion in rats. Free Radical Research, 50, 645–653. 26953687 10.3109/10715762.2016.1162300

[phy214297-bib-0021] Kaludercic, N. , Carpi, A. , Menabò, R. , Di Lisa, F. , & Paolocci, N. (2011). Monoamine oxidases (MAO) in the pathogenesis of heart failure and ischemia/reperfusion injury. Biochimica Et Biophysica Acta, 1813, 1323–1332. 20869994 10.1016/j.bbamcr.2010.09.010 PMC3030628

[phy214297-bib-0022] Lesch, K. P. , Wolozin, B. L. , Murphy, D. L. , & Reiderer, P. (1993). Primary structure of the human platelet serotonin uptake site: Identity with the brain serotonin transporter. Journal of Neurochemistry, 60, 2319–2322. 7684072 10.1111/j.1471-4159.1993.tb03522.x

[phy214297-bib-0023] Li, R. W. , Yang, C. , Kwan, Y. W. , Chan, S. W. , Lee, S. M. , & Leung, G. P. (2013). Involvement of organic cation transporter‐3 and plasma membrane monoamine transporter in serotonin uptake in human brain vascular smooth muscle cells. Frontiers in Pharmacology, 4, 14. 23407616 10.3389/fphar.2013.00014 PMC3569667

[phy214297-bib-0024] Longhurst, J. C. , Tjen‐A‐Looi, S. C. , & Fu, L. W. (2001). Cardiac sympathetic afferent activation provoked by myocardial ischemia and reperfusion: mechanisms and reflexes. Annals of the New York Academy of Sciences, 940, 74–95. 11458709 10.1111/j.1749-6632.2001.tb03668.x

[phy214297-bib-0025] Métais, C. , Bianchi, C. , Li, J. , Simons, M. , & Sellke, F. W. (2001). Serotonin‐induced human coronary microvascular contraction during acute myocardial ischemia is blocked by COX‐2 inhibition. Basic Research in Cardiology, 96, 59–67. 11215533 10.1007/s003950170078

[phy214297-bib-0026] Mortensen, O. V. , Kristensen, A. S. , Rudnick, G. , & Wiborg, O. (1999). Molecular cloning, expression and characterization of a bovine serotonin transporter. Molecular Brain Research, 71, 120–126. 10407194 10.1016/s0169-328x(99)00178-3

[phy214297-bib-0027] Pizzinat, N. , Copin, N. , Vindis, C. , Parini, A. , & Cambon, C. (1999). Reactive oxygen species production by monoamine oxidases in intact cells. Naunyn‐Schmiedeberg's Archives of Pharmacology, 359, 428–431. 10.1007/pl00005371 10498294

[phy214297-bib-0028] Sari, Y. , & Zhou, F. C. (2003). Serotonin and its transporter on proliferation of fetal heart cells. International Journal of Developmental Neuroscience, 21, 417–424. 14659992 10.1016/j.ijdevneu.2003.10.002

[phy214297-bib-0029] Schömig, E. , Babin‐Ebell, J. , & Russ, H. (1993). 1,1'‐diethyl‐2,2'‐cyanine (decynium22) potently inhibits the renal transport of organic cations. Naunyn‐Schmiedeberg's Archives of Pharmacology, 347, 379–383. 10.1007/BF00165387 8510766

[phy214297-bib-0030] Shimizu, Y. , Minatoguchi, S. , Hashimoto, K. , Uno, Y. , Arai, M. , Wang, N. , … Fujiwara, H. (2002). The role of serotonin in ischemic cellular damage and the infarct size‐reducing effect of sarpogrelate, a 5‐hydroxytryptamine‐2 receptor blocker, in rabbit hearts. Journal of the American College of Cardiology, 40, 1347–1355. 12383585 10.1016/s0735-1097(02)02158-7

[phy214297-bib-0031] Solbach, T. F. , Grube, M. , Fromm, M. F. , & Zolk, O. (2011). Organic cation transporter 3: Expression in failing and nonfailing human heart and functional characterization. Journal of Cardiovascular Pharmacology, 58, 409–417. 21697722 10.1097/FJC.0b013e3182270783

[phy214297-bib-0032] Sonobe, T. , Akiyama, T. , Du, C. K. , Zhan, D. Y. , & Shirai, M. (2013). Contribution of serotonin uptake and degradation to myocardial interstitial serotonin levels during ischaemia‐reperfusion in rabbits. Acta Psychologica, 207, 260–268. 10.1111/j.1748-1716.2012.02461.x 22687057

[phy214297-bib-0033] Sonobe, T. , Akiyama, T. , Du, C. K. , Zhan, D. Y. , & Shirai, M. (2014). Contribution of calpain to myoglobin efflux from cardiomyocytes during ischaemia and after reperfusion in anaesthetized rats. Acta Psychologica, 210, 823–831. 10.1111/apha.12205 24256333

[phy214297-bib-0034] Wang, J. (2016). The plasma membrane monoamine transporter (PMAT): Structure, function, and role in organic cation disposition. Clinical Pharmacology and Therapeutics, 100, 489–499. 27506881 10.1002/cpt.442 PMC5305120

[phy214297-bib-0035] Willerson, J. T. , Golino, P. , Eidt, J. , Campbell, W. B. , & Buja, L. M. (1989). Specific platelet mediators and unstable coronary artery lesions. Experimental evidence and potential clinical implications. Circulation, 80, 198–205. 10.1161/01.CIR.80.1.198 2661053

